# Rethinking Slovenia’s health technology assessment system

**DOI:** 10.1017/S0266462326103948

**Published:** 2026-07-09

**Authors:** Rok Hren

**Affiliations:** 1 Syreon Research Institute, Budapest, Hungary; 2 Institute of Mathematics, Physics and Mechanics, Ljubljana, Slovenia

I read with great interest the recent article by Beravs-Bervar et al., “Strengthening health technology assessment (HTA) in the European Union: Insights from Slovenia’s implementation journey.” (*Int J Technol Assess Health Care.* 2026;42(1):e23. https://doi.org/10.1017/S0266462325103383). The article offers an overview of the institutional challenges Slovenia faces in aligning with the EU HTA Regulation, and to that end, fragmentation, limited methodological capacity, and the absence of a formally designated HTA body can indeed be seen as areas for further development (1).

However, the overall characterization of the Slovenian system as “underdeveloped” risks being overly reductive and insufficiently nuanced. In particular, the manuscript appears to underrecognize a key strength of the Slovenian healthcare system, the well-functioning and highly pragmatic pricing and reimbursement (P&R) framework for medicinal products. This framework has demonstrably enabled rapid and equitable access to innovative therapies, placing Slovenia on a par with some of the most advanced jurisdictions globally (2).

As acknowledged in the article, medicinal products in Slovenia are already subject to structured evaluation incorporating clinical effectiveness, cost-effectiveness, budget impact, and ethical considerations (1;3). What distinguishes the Slovenian model is not the absence of HTA principles, but rather their integration within a streamlined, single-payer decision-making process, avoiding institutional duplication and enabling timely access, which is an especially important feature in a small healthcare system with limited human resources (2).

While the authors advocate for the establishment of a centralized HTA body, the implicit assumption that increased institutionalization will necessarily improve outcomes warrants careful consideration. Experience from Central and Eastern Europe suggests that greater procedural complexity does not automatically translate into better access, and may, in some cases, delay patient access to innovation (2;4). In this context, future reforms should aim not only to align with EU requirements but also to preserve the strengths of the existing system, particularly its efficiency, pragmatism, and outcome-oriented approach.
